# Circular RNA expression profile in the spinal cord of morphine tolerated rats and screen of putative key circRNAs

**DOI:** 10.1186/s13041-019-0498-4

**Published:** 2019-09-18

**Authors:** Yingqi Weng, Jing Wu, Lin Li, Jiali Shao, Zhengyiqi Li, Meiling Deng, Wangyuan Zou

**Affiliations:** 1 Department of Anesthesiology, Xiangya Hospital, Central South University, Changsha, 410008 Hunan China; 2 Department of Anesthesiology, The First Hospital of Changsha, Changsha, 410008 Hunan China; 3 Department of Anesthesiology, Hunan Cancer Hospital, Central South University, Changsha, 410008 Hunan China; 4 National Clinical Research Center for Geriatric Disorders, Xiangya Hospital, Central South University, Changsha, 410008 Hunan China

**Keywords:** Morphine tolerance, circRNA, ceRNA

## Abstract

Morphine tolerance developed after repeated or continuous morphine treatment is a global health concern hindering the control of chronic pain. In our previous research, we have reported that the expression of lncRNAs and microRNAs have been greatly modified in the spinal cord of morphine tolerated rats, and the modulating role of miR-873a-5p, miR-219-5p and miR-365 have already been confirmed. However, whether circular RNAs, another essential kind of non-coding RNA, are involved in the pathogenesis of morphine tolerance is still beyond our knowledge. In this study, we conducted microarray analysis for circRNA profile and found a large number of circRNAs changed greatly in the spinal cord by morphine treatment. Among them, we selected nine circRNAs for validation, and seven circRNAs are confirmed. Gene Ontology/Kyoto Encyclopedia of Genes and Genomes (GO/KEGG) analysis were used for functional annotation. Besides, we confirmed the modified expression of seven circRNAs after validation by real-time PCR, selected 3 most prominently modulated ones among them and predicted their downstream miRNA-mRNA network and analyzed their putative function via circRNA-miRNA-mRNA pathway. Finally, we enrolled the differentially expressed mRNAs derived from the identical spinal cord, these validated circRNAs and their putative miRNA targets for ceRNA analysis and screened a promising circRNA-miRNA-mRNA pathway in the development of morphine tolerance. This study, for the first time, provided valuable information on circRNA profile and gave clues for further study on the circRNA mechanism of morphine tolerance.

## Introduction

Morphine is widely used in the management of acute and chronic pain, and remains among the most effective drugs for moderate and severe pain and escalated cancer pain nowadays. Besides multiple side effects, a major problem that hinders its use is the analgesic tolerance developed after repeated or continuous utility [1–3]. For decades, a large number of studies at the molecular, cellular, and systems levels have been devoted to elucidating the underlying mechanism. The researchers have already determined the involvement of dissociation between opioid receptor-G protein-coupled receptors, increased internalization and disrupted recycling of MOR, β-arrestin-2 mediated MOR desensitization, MOR and δ-OR interaction, PKA/PKC and MAPKs pathway activation in the processes leading to morphine tolerance [3–9]. However, we are far away from fully understanding of the mechanisms underlying this phenomenon. Meanwhile, morphine tolerance is barely prevented effectively. According to the analyses of the human transcriptome, most transcripts are identified as non-coding RNAs with little or no protein-coding capacity, and this provides new scene out of traditional protein-centric molecular biology [10]. MicroRNAs (miRNAs), long non-coding RNAs (lncRNAs) and circular RNAs (circRNAs) are the most acknowledged components of non-coding RNAs acting as epigenetic regulators. Under many pathological conditions in the nervous system, they have been found to be greatly modified and are suggested to be promising biomarkers and therapeutic targets. For example, Gao’s group has reported that hundreds of lncRNAs and mRNAs were dysregulated in the spinal cord of mice with neuropathic pain induced by spinal nerve ligation [11]. In our previous research, we have identified that plenty of microRNAs and lncRNAs were dramatically modified in the lumbar spinal cord of rats with morphine tolerance, suggesting the non-coding RNAs may have extensive effect in this condition [12–14]. As a unique type of RNAs which is distinguished from widely-known linear RNA, circular RNAs are back-spliced from exons, introns or both, with the 3′ and 5′ ends joined together to form a covalently closed continuous loop [15]. CircRNAs have been discovered for decades but were misinterpreted as useless splicing errors [16]. In 2013, they were rediscovered to be widespread and diverse in eukaryotic cells by RNA sequencing (RNA-seq) [17]. Though recently circRNAs have been documented to encode proteins, most studies reveal their major role as non-coding RNAs exerting transcriptional and post-transcriptional regulatory effects [18–20]. Similar to other non-coding RNAs, circRNAs have been considered valuable in understanding the pathogenesis of diseases and developing diagnostic biomarkers for diseases [21].

To reveal the potential roles of circRNAs in the process of morphine tolerance, we first identified dysregulated circRNAs in the spinal cord of morphine tolerant rats via microarray analysis. Then we validated a few circRNAs that were up- or down-regulated in the spinal cord by real-time PCR, and predicted their function using bio-informative methods. This research provided clues for further research on the regulatory network of non-coding RNAs in the etiology of morphine tolerance and on the exploration of novel targets for the treatment or prevention.

## Materials and methods

### Repeated intrathecal injection of morphine induces morphine tolerance

We used the identical spinal cord samples for circRNA, lncRNA and mRNA analysis, thus the establishment of morphine tolerated rat model was the same as we described in our previous research [12]. In brief, adult male Sprague-Dawley rats in the morphine tolerance group (MT group, *n* = 8) received 10 μg intraperitoneal morphine (1 μg/1 μL) twice a day at 08:00–09:00 am and 4:00~5:00 pm for 7 consecutive days [2]. Their cohorts in the normal saline group (NS group, n = 8) received equal volumes of normal saline following the identical protocol. The tail-flick test utilizing Hargreaves apparatus (Italy, UGO Basile) was applied to examine the thermal sensitivity of rats. The results were converted to the maximum possible effect (%MPE) to evaluate the effect of morphine and confirm the establishment of morphine tolerance.

### RNA extraction and quality control

On the 8th day, one hour after the injection with morphine or saline in the morning, the rats were decapitated under deep anesthesia by pentobarbital sodium (1%). The lumbar enlargements were collected on ice and snap-frozen in liquid nitrogen. The RNA isolation was performed by Kangcheng Bio-tech (Shanghai, China) using TRIzol reagent (Invitrogen, Carlsbad, CA, USA) and following the manufacturer’s protocol. The purity and concentration of total RNA were determined with NanoDrop ND-1000 (NanoDrop, Wilmington, DE, USA). The RNA integrity was assessed by denaturing agarose gel electrophoresis. The remnant RNA was stored for later use at − 80 °C.

### CircRNA microarrays

Sample labeling and array hybridization were performed following the manufacturer’s protocol (Arraystar Inc.). Briefly, total RNAs were digested with Rnase R (Epicentre, Inc.) to get rid of the linear RNAs, thus the circular RNAs were enriched. Then, by using the random priming method (Arraystar Super RNA Labeling Kit; Arraystar), we purified the circular RNAs and transcribed them into fluorescent cRNA. After purifying the labeled cRNAs (RNeasy Mini Kit, Qiagen), we measured the concentration and specific activity of these labeled cRNAs (pmol Cy3/μg cRNA) by NanoDrop ND-1000. 5 μl 10 × Blocking Agent and 1 μl of 25 × Fragmentation Buffer were added into 1 μg of each labeled cRNA for fragmentation, then the mixture was heated at 60 °C for 30 min. Finally, 25 μL 2 × Hybridization buffer was added for dilution. The labeled cRNAs were hybridized onto the Arraystar Rat circRNA Array (8 × 15 K, Arraystar). After incubation for 17 h at 65 °C in an Agilent Hybridization Oven, the hybridized arrays were washed, fixed and scanned using the Agilent Scanner G2505C.

### CircRNA data analysis and bioinformatics

Raw data (uploaded to the GEO database as GSE133602) were extracted from the imported scanned images by Agilent Feature Extraction software (version 11.0.1.1) then processed using R software limma package. Low intensity filtering was performed after quantile normalization of the raw data. In conformity with the definitions and instructions in GeneSpring software, circRNAs with at least 4 out of 8 samples that have flags in “P” or “M” (“All Targets Value”) were retained for further analysis. Fold change was computed and Student’s t-test with Benjamini-Hochberg multiple testing correction for false discovery rate was performed between two groups (morphine tolerance versus normal saline) for circRNA filtration. CircRNAs exhibiting fold changes ≥2.0 and *p*-values ≤0.05 were selected as significantly differentially expressed circRNAs. The functional classification and significant pathways of the circRNA parent genes were conducted using Gene Ontology (www.geneontology.org) and the latest Kyoto Encyclopedia of Genes and Genomes (KEGG) database (www.genome.jp/kegg). Arraystar’s home-made miRNA target prediction software, which is based on TargetScan and miRanda, was used to predict circRNAs-targeted miRNAs. The circRNAs expression profile microarray chip assay, data analysis and bioinformatics were carried out by KangChen Bio-tech, Shanghai.

### qRT-PCR assay

Nine circRNAs were selected for validating differentially expressed circRNAs by utilizing real-time PCR. Briefly, total RNAs extracted from the spinal tissue samples of two groups (*n* = 3 or 4 for each group) were reverse-transcribed into cDNA with SuperScript™ III Reverse Transcriptase (Invitrogen). Quantitative RT-PCR was conducted in the ViiA 7 Real-time PCR System (Applied Biosystems) using PCR master mix (Arraystar). Glyceraldehyde 3-phosphate dehydrogenase (GAPDH) were quantified as internal controls for data normalization. The sequences of all primers are presented in Additional file 1: Table S1.

### Function prediction of validated differentially-expressed circRNAs (DEcircRNAs)

CircRNA contains multiple binding sites to miRNAs that lead to the sponge interaction between circRNA and miRNAs [22, 23]. The circRNAs/miRNA interaction was predicted using home-made miRNA target prediction software of Arraystar based on TargetScan and miRanda. The miRNA and mRNA interaction was predicted based on miRDB database (http://www.mirdb.org). For each validated circRNA, five putative target miRNAs with the highest matching score were identified according to the analysis mentioned above, thus 15 miRNAs in total were used for their target mRNA prediction and miRNA/mRNA interacting network generation. Then these mRNAs were submitted for GO and KEGG analysis to estimate the possible function of circRNAs via interacting with miRNAs.

### CeRNA analysis

As we have already filtered a large amount of differentially expressed mRNAs in the identical spinal cord samples in our previous research, we enrolled the validated DEcircRNAs, all of their predicted target miRNAs and the differentially expressed mRNAs for ceRNA analysis and generated the circRNA/miRNA/mRNA interaction network using Cytoscape software.

### Statistical analysis

All data were presented as mean ± s.e.m. The real-time PCR data fulfilled the assumption of normal distribution and equal variances, so the comparison between two groups was conducted using unpaired Student’s t-test. *P* values less than 0.05 were considered statistically significant.

## Results

### Construction of the rat morphine tolerance model

We used the identical spinal cord tissue of rats for circRNA, lncRNA and mRNA microarray analysis. Thus, as previously reported, the morphine tolerated rat model was constructed successfully after repeated intrathecal injection of morphine for consecutive seven days when the MPE% almost dropped to 0 [12].

### Profiling of circRNAs in the spinal cord of rats with morphine or saline injection

**Fig. 1:**
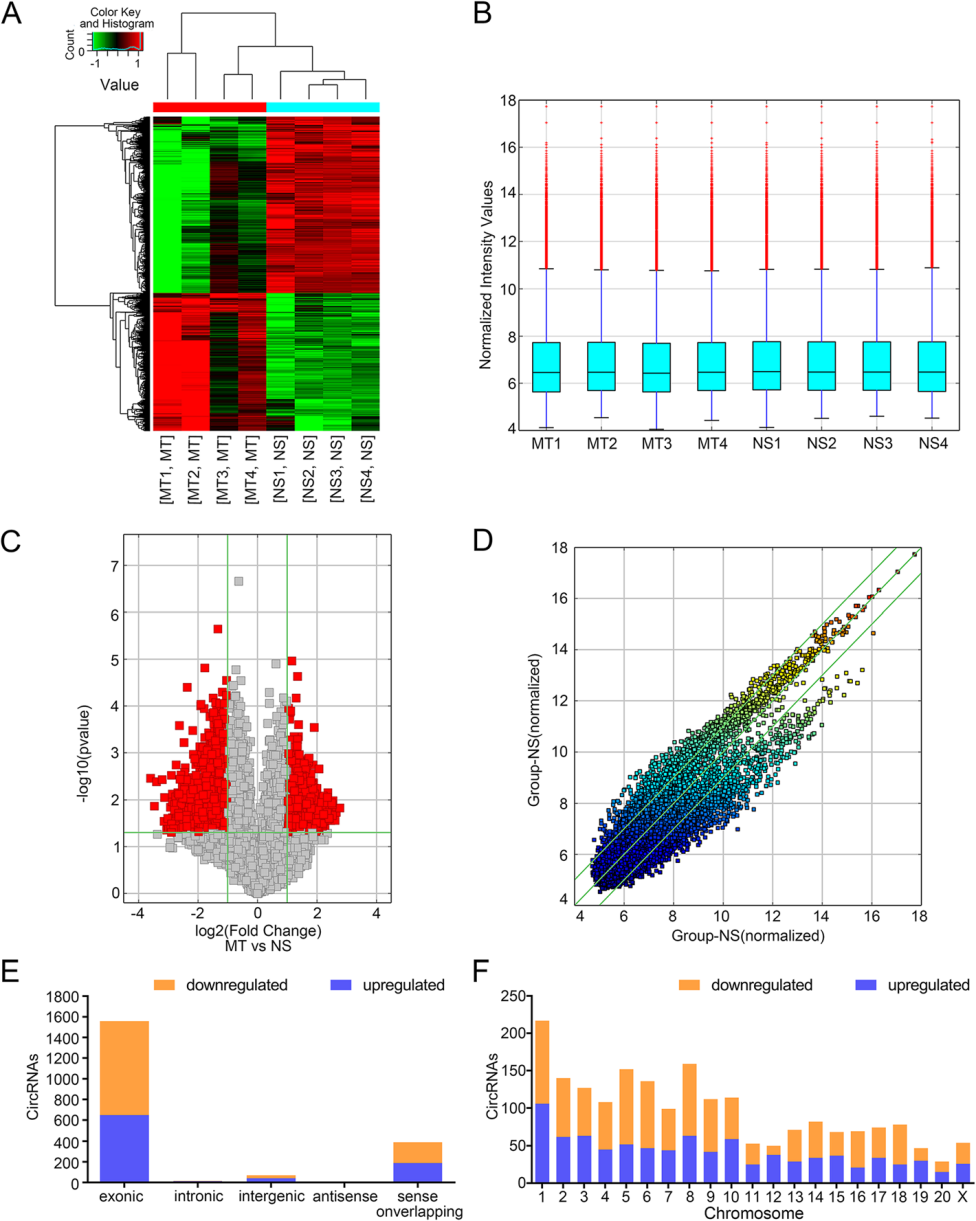
The circRNA expression profile in the spinal cord of morphine-tolerated and sham rats. **a**. Heat map generated by hierarchical clustering of differentially expressed circRNAs in 4 MT and 4 NS samples; the highly- and lowly-expressed circRNAs are represented in red and green respectively; **b**. The box plot shows the enrichment of total circRNAs in each sample; **c**. The red spots in the volcano plot represented the differentially expressed circRNAs with statistical significance; **d**. Scatter plot illustrated the normalized circRNA expression in both groups. The x-axis represented the circRNA level in NS group, the y-axis represented the circRNA level in MT group, circRNAs distributed above the top green line or below the green bottom line are the ones with a between-group fold change more than 2.0; **e**. The classification of DEcircRNAs based on their origin, most DEcircRNAs were originated from exons. **f**. The chromosome distribution of DEcircRNAs: the host genes of DEcircRNAs were distributed in all chromosomes. MT, morphine tolerance; NS, normal saline

According to the filtration criteria (fold changes ≥2.0 and *p*-values ≤0.05), there were 2038 circRNAs differentially expressed between MT and NS group, consisting of 896 up-regulated and 1142 circRNAs down-regulated circRNAs. All these DEcircRNAs were displayed in the hierarchical clustering in Fig. 1a, with red color representing high read counts and green color representing low read counts of circRNAs. The enrichment of total circRNAs in the spinal cord tissue from 4 MT rats and 4 NS rats was estimated and illustrated in Fig. 1b. As demonstrated in the volcano plot in Fig. 1c, the up-regulated circRNAs were represented as the red dots on the left, while the down-regulated circRNAs were represented as the red dots on the right. The average level of each circRNA in both groups was shown in the scatter plot in Fig. 1d, it is obvious that a large number of circRNAs were significantly modified by morphine treatment. Thirty mostly increased and mostly decreased circRNAs were listed in Additional file 2: Table S2. Most of the host genes encoding the DEecircRNAs are exonic, and they distribute over all chromosomes (Fig. 1e-f). All of the microarray results have been uploaded to the GEO database (GSE133602).GO and KEGG analysis of the biological function of circRNA host genes.

**Fig. 2:**
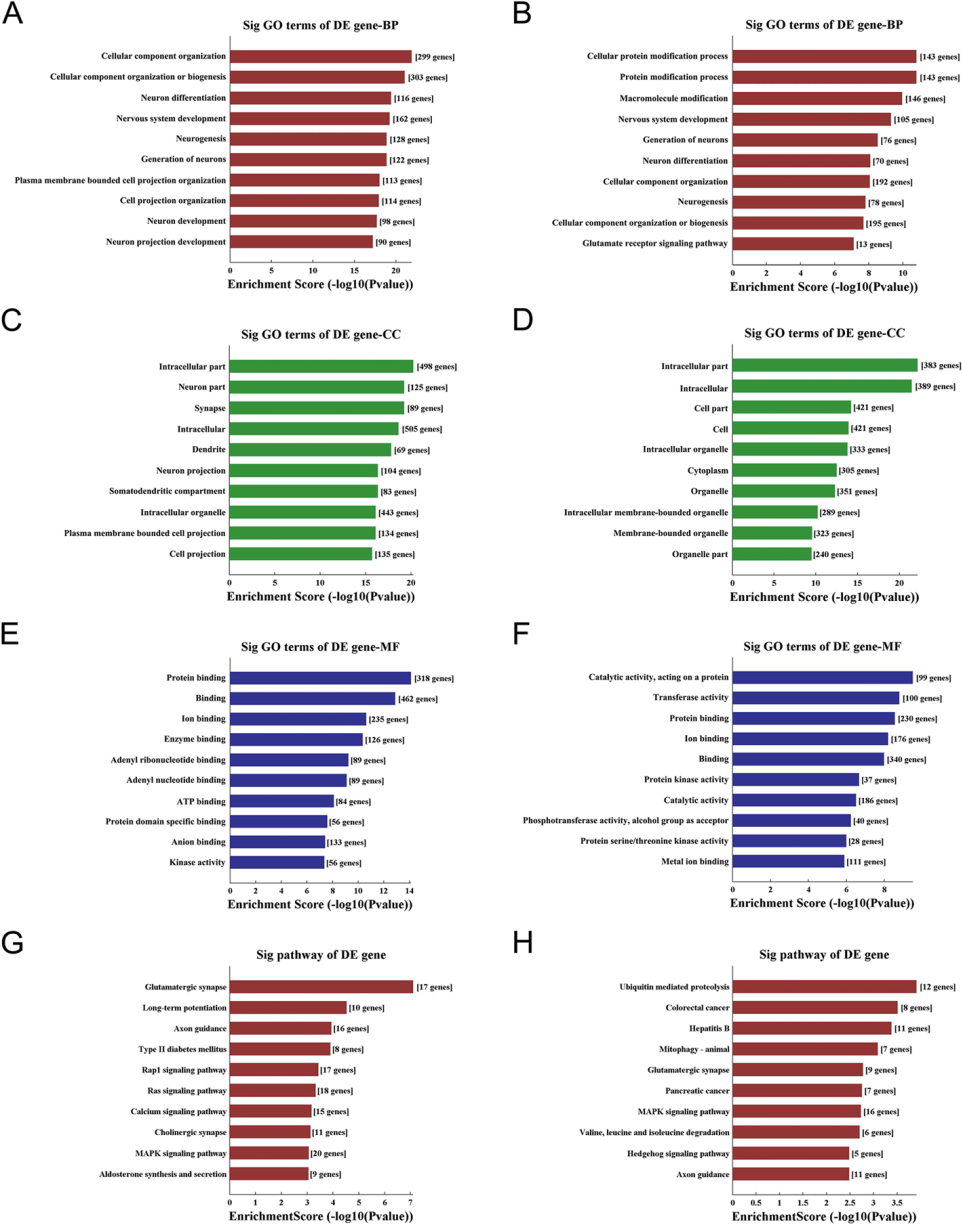
Gene Ontology and KEGG Pathway enrichment for the host genes encoding DEcircRNAs. The left column (**a**, **c**, **e** and **g**) and right column (**b**, **d**, **f** and **h**) represented the result of up- and down-regulated circRNAs, respectively. BP: biological process, CC: cellular component, and MF: molecular function

The host genes of these DEcircRNA were submitted for GO and KEGG analysis. The result of GO analysis for the up and down-regulated circRNAs respectively was listed in Fig. 2a-f, consisting of three different aspects named biological process (BP), cellular component (CC) and molecular function (MF). According to the KEGG analyses, the most significantly enriched pathways of the up-regulated circRNA host genes were glutamatergic synapse, long-term potentiation, axon guidance, type II diabetes mellitus, Rap1 signaling pathway, Ras signaling pathway, calcium signaling pathway, cholinergic synapse, MAPK signaling pathway and aldosterone synthesis and secretion (Fig. 2g). While the most significantly enriched pathways of the down-regulated circRNA host genes were ubiquitin-mediated proteolysis, colorectal cancer, hepatitis B, mitophagy, glutamatergic synapse, pancreatic cancer, MAPK signaling pathway, valine, leucine and isoleucine degradation, hedgehog signaling pathway and axon guidance (Fig. 2h).

### Validation of DEcircRNA by real-time PCR

**Fig. 3:**
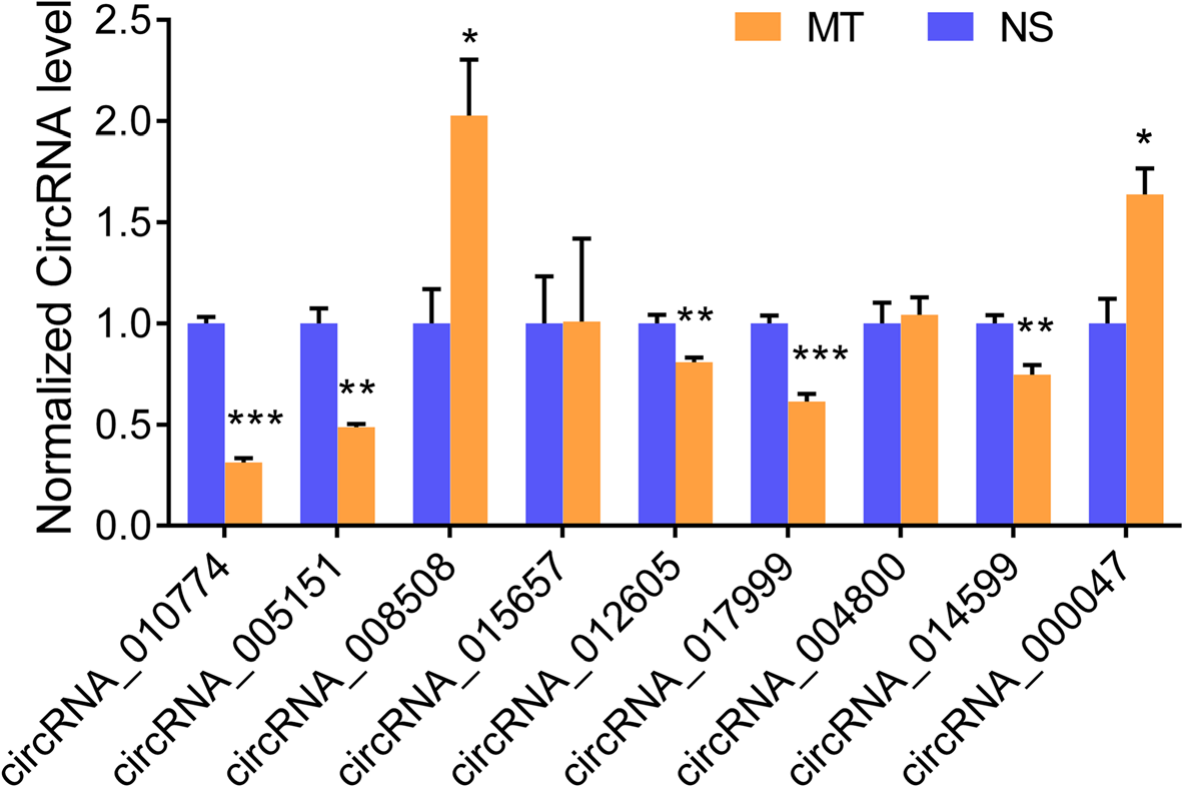
Verification of selected DEcircRNAs expression by real-time PCR. NS, normal saline; MT, morphine tolerance. ^*^P<0.05; ^**^P<0.01; ^***^P<0.001, NS vs. MT

Four up-regulated and five down-regulated circRNAs with great expression difference and small *p*-value between groups, high abundance and low intra-group variation were selected for validation. As what was shown in Fig. 3, the rno_circRNA_005151, rno_circRNA_010774, rno_circRNA_014599, rno_ circRNA_012605 and rno_circRNA_017999 were significantly down-regulated, the rno_circRNA_008508 and rno_circRNA_000047 were significantly up-regulated in morphine tolerated rats, consistent with the microarray result, but the expression of rno_circRNA_015657 and rno_circRNA_004800 kept unchanged between two groups.

### Function prediction of the validated DEcircRNAs

**Fig. 4:**
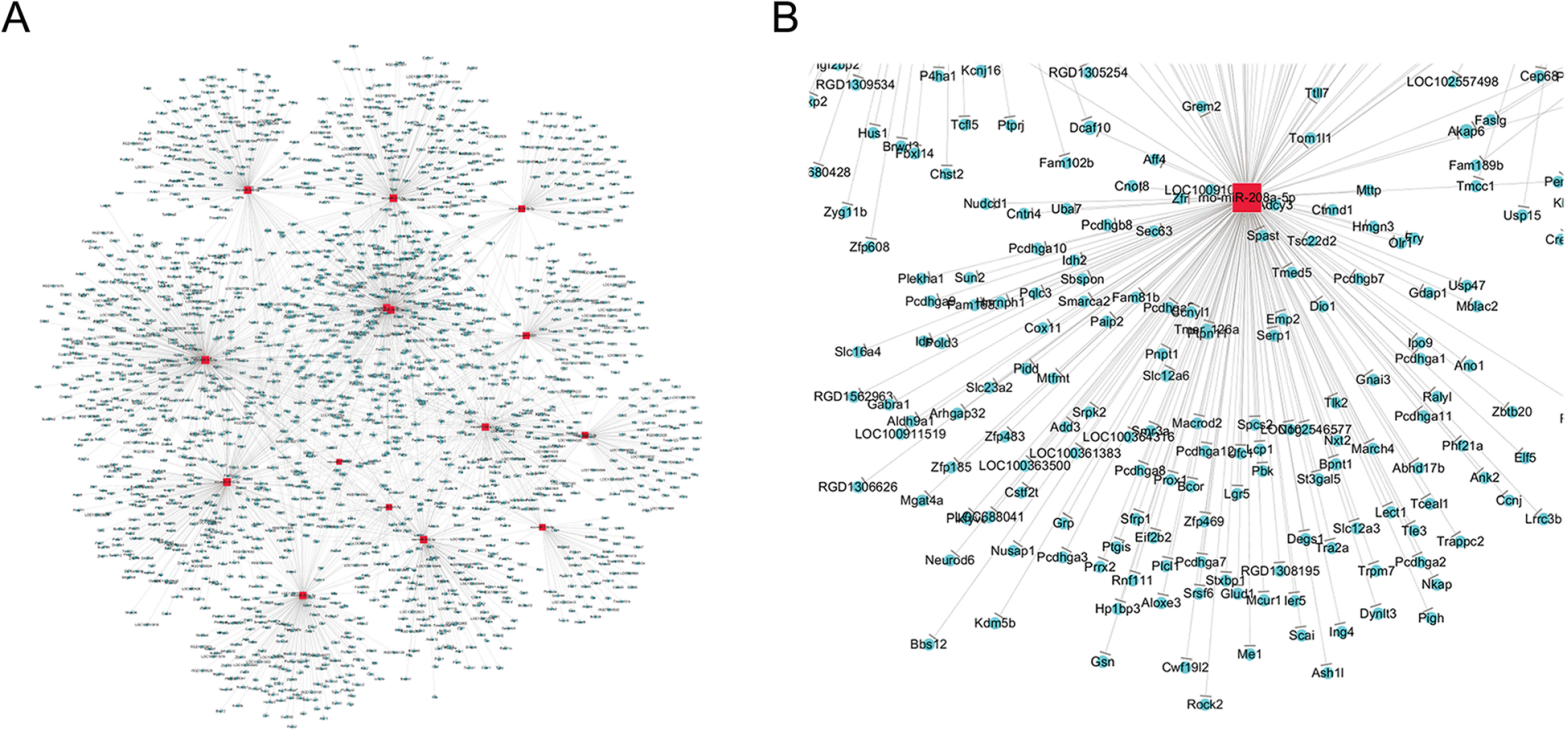
The predicted downstream miRNA-mRNA network of the validated DEcircRNAs. **a**. the panorama of the miRNAs-mRNA network; **b**. the partially enlarged detail of the miRNAs-mRNA network; red and blue spots represented miRNA and mRNA respectively

**Fig. 5:**
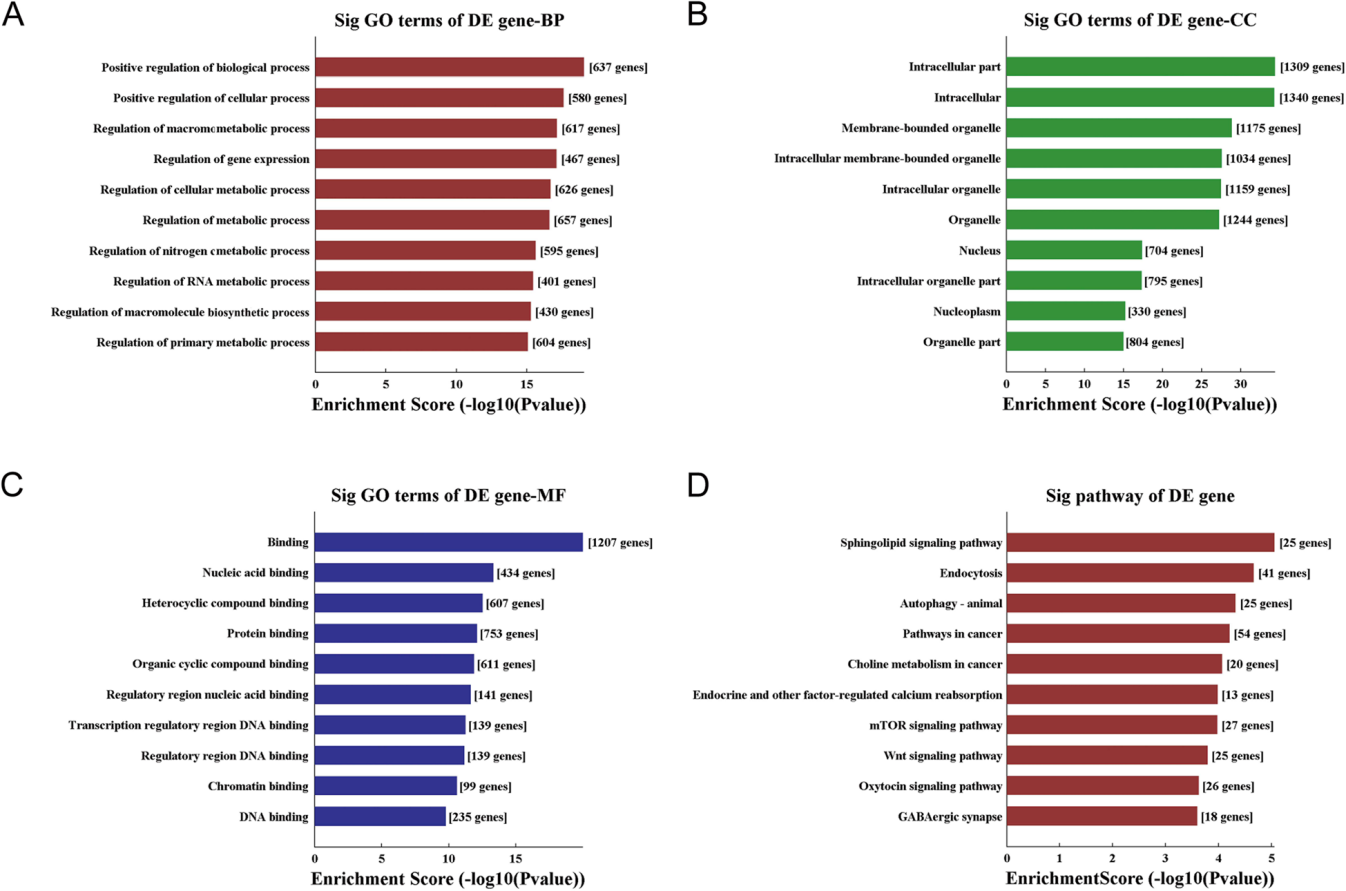
Gene Ontology (**a**-**c**) and KEGG Pathway enrichment (**d**) for the putative target genes of validated DEcircRNAs via circRNA/miRNAs pathway

Among these validated DEcircRNAs,rno_circRNA_005151, rno_circRNA_010774 and rno_circRNA_015657 were selected for further function prediction. For each one of them, five putative interacting miRNAs with the highest matching score were selected (listed in Additional file 3: Figure S1). Then, these 15 miRNAs in total were used for prediction of their target mRNAs and a network containing these miRNAs and genes was constructed as shown in Fig. 4. The result of GO and KEGG analysis of these target genes that may be modulated through circRNA-miRNA-mRNA pathways was exhibited in Fig. 5. The most highly enriched biological process, cellular component and molecular function were regulation of gene expression, intracellular part and binding, respectively (Fig. 5a-c). The most significantly enriched pathways of these target genes were sphingolipid signaling pathway, endocytosis, autophagy, pathways in cancer, choline metabolism in cancer, endocrine and other factor-regulated calcium reabsorption, mTOR signaling pathway, Wnt signaling pathway, oxytocin signaling pathway, GABAergic synapse (Fig. 5d).

### CeRNA analysis for the validated DEcircRNAs

**Fig. 6:**
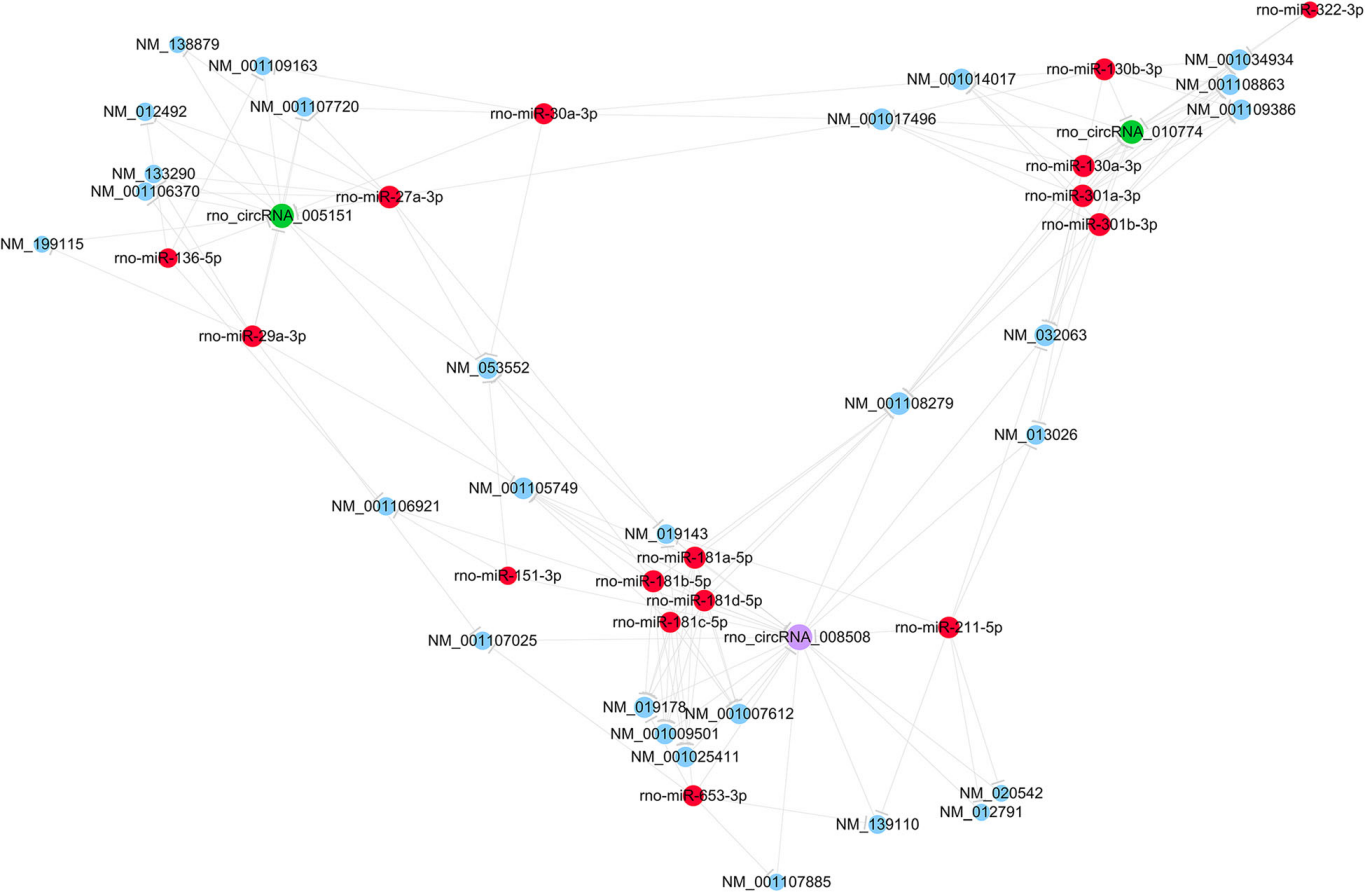
CeRNA analysis for the validated DEcircRNAs, putative target miRNAs and DEmRNAs. Green and purple spots represented down- and up-regulated circRNAs respectively, red and blue spots represented putative target miRNAs and microarray-filtered DEmRNAs respectively

In our previous research, we have filtered plenty of differentially-expressed mRNAs (DEmRNAs) in the identical spinal cord tissues [12]. The interactions between the three selected circRNAs after validation, all of their predicted interacting miRNAs and these DEmRNAs were calculated and visualized as the CeRNA network as Fig. 6.

## Discussion

In this study, we reported circRNA profiles in the central nervous system of morphine-tolerated rats for the first time. The profile revealed that 896 circRNAs were up-regulated and 1142 circRNAs were down-regulated in the lumbar spinal cord tissue after tolerance induction by chronic morphine treatment. Also, we validated three DEcircRNAs, then constructed their putative downstream miRNAs-mRNA network based on the circRNA-miRNA and miRNA-mRNA interaction prediction. The GO and KEGG analysis according to the host gene of all microarray-screened DEcircRNA and the genes under putative modulation by validated circRNAs via circRNA/miRNA/mRNA pathways provided an overview for the role of circRNAs in morphine tolerance. Finally, in combination with the DEmRNAs data derived from our previous research, we conducted the ceRNA analysis and constructed a circRNA/miRNA/mRNA interaction network [12].

A large number of actively transcribed human genes have been reported producing circRNAs [24, 25]. The well recognized biogenetic process of circRNAs requires repetitive element sequences to form intronic complementary sequences (ICSs) in the introns flanking circularized exons [23]. However, there are also other unknown back-splicing mechanisms, as in our finding, circ_005151 includes the first exon of WHSC1, which was usually not believed to be circularized because of the lack of upstream intron. The similar finding was also reported by Kristensen et al., who noticed the circRNA from the KRT5 gene is derived from cryptic splice sites found within the first exon [26]. And according to the circBase, there are 4 circRNAs may be originated from KRT5 contain the first exon.

It is now widely accepted that circRNAs could be expressed dynamically and play an indispensable role during cell cycle, development, organ differentiation, and upon pathological conditions [27, 28]. CircRNAs could exert their function through multiple mechanisms. A major one is modulating their host genes by regulating splicing or transcription and by interacting with RNA-binding proteins (RBPs) [29, 30]. Resultantly, though controversies keep existing, most researchers reported that the circRNAs level and their linear counterpart from the same gene were largely correlated [17, 27, 31]. Based on this, we first conducted the GO analysis and KEGG pathway analysis for the host genes of the DEcircRNAs to predict the putative functions of them. The GO analysis implied that the DEcircRNAs were presumably involved in the development and differentiation of neurons and synapses, the development of the nervous system and transmission of neural signals. The KEGG pathway analysis showed enrichment in the biological processes of glutamatergic synapse, MAPK signaling pathway and axon guidance. It could be reasonable, as many of these processes are considered accounting for the formation of chronic morphine tolerance. After chronic exposure to morphine, the phosphorylation of ERK and p38, two essential components of the MAPK family, was up-regulated. Inhibition of ERK and p38 was able to attenuate the analgesic tolerance of morphine [8, 32–36]. A study proposed one of the possible mechanism that morphine activates MOR/AKT/K_ATP_/ERK pathway to induce the HSP70 release from the neuron, which activated microglia and led to p38 and NF-κB p65 phosphorylation, activation of NLRP3 inflammasome and analgesic tolerance finally [37]. Besides modulating MAPK pathway, chronic morphine exposure significantly increases the expression and activity of the glutamatergic receptor, enhances glutamatergic synaptic transmission, and down-regulates the membrane glutamate transporters GLT-1 in the spinal cord, hippocampus or nucleus raphe magnus respectively [38–41]. Blocking the spinal NMDAR, a critical pre- and post-synaptic glutamatergic receptor, significantly attenuated the development of morphine tolerance [42, 43].

Another essential function of circRNAs is serving as a sponge to miRNAs [22, 23]. Thus, for the validated DEcircRNAs, we predicted their interactive miRNAs with the highest matching score, then enrolled the putative downstream target mRNAs for GO and KEGG pathway analysis. The annotation of these target genes gave clues for the function of the validated circRNAs through circRNA/microRNA/mRNA pathways. Consistent with our prediction, in the spinal cord, the involvement of GABAergic synapse, mTOR signaling pathway and autophagy in morphine tolerance has already been documented. Chronic morphine treatment induces CatB-dependent excessive autophagy and leads to GABAergic interneurons dysfunction in the superficial layer of the spinal cord. Blocking autophagy or CatB could successfully inhibit the development of morphine tolerance dose-dependently [44]. Another study reported an increased abundance of GABA transporter 1(GAT-1), which regulates the level of GABA, in the lumbar spinal cord after chronic morphine consumption. Inhibition of GAT-1 improves the antinociceptive effect of morphine [45]. μ opioid receptor activation triggers the PI3K/Akt/mTOR pathway to promotes spinal protein translation and finally leads to morphine tolerance and hyperalgesia [46].

In our previous research, we have already screened a great number of mRNAs that were greatly changed in the identical spinal cord samples [12]. By computative ceRNA analysis utilizing the validated DEcircRNAs, all of their putative target miRNAs and the microarray-filtered DEmRNAs, we noticed that the up-regulated circRNA_008508 was able to suppress miR-181b-5p, miR-181d-5p, miR-181c-5p and miR-181a-5p, while the miR-181 family could bind to Toll-like receptor 4 (TLR-4) according to the prediction. As proved by other researchers, miR-181b and miR-181c suppressed TLR-4 expression directly [47, 48]. Chronic morphine treatment evoked activation of toll-like receptor 4 (TLR4) in microglia, which led to NOD-like receptor protein 3 (NLRP3) inflammasome and NF-κB activation, enhanced proinflammatory cytokines such as TNF-α and IL-1β, and resultantly facilitated the development and maintenance of analgesic tolerance [37, 49]. So, it is reasonable to assume that the elevated circRNA_008508 may sponge with miR-181 family and relieve their suppression to TRL-4, thus evoke neuroinflammation and promote morphine tolerance finally. This hypothesis will be tested in our further study. With all these inspiring functional predictions of DEcircRNAs, it is noticeable that the stoichiometric analyses will be important when confirming the miRNA “sponge” effect to any RNAs. The stoichiometric analyses quantify the abundance of miRNAs and their RNA binding sites, which influences the competition between target sites and finally shapes the effect of miRNAs along with other elements such as the affinity of binding sites [50].

Though the change of over 2000 circRNAs was detected by microarray analysis, their function has barely been identified in vivo or in vitro. The interaction between circRNAs and miRNAs has been solely predicted by software, further validation should be made in future studies. Even though with many questions not answered, it is the first study conducting circRNA profile and ceRNA analysis in the morphine-tolerated model, it will provide valuable information for exploring the role of non-coding RNAs in the pathogenesis of morphine tolerance.

## Supplementary information

**Additional file 1: Table S1.** The primers for real-time PCR.

**Additional file 2: Table S2.** The detailed information of top 30 up-regulated and 30 down-regulated circRNAs.

**Additional file 3: Figure S1.** The putative target miRNAs of the validated circRNAs with the highest matching score. A-C. Putative target miRNAs of circRNA _ 005151, _008508 and _010774 respectively.

## Data Availability

Please contact the author for data requests.

## Abbreviations

BP: Biological process
CC: Cellular component
circRNA: Circular RNA
DEcircRNAs: Differentially-expressed circRNAs
DEmRNAs: Differentially-expressed mRNAs
GO: Gene Ontology
KEGG: Kyoto Encyclopedia of Genes and Genomes
lncRNA: Long non-coding RNA
MF: Molecular function
miRNA: MicroRNA
qRT-PCR: Quantitative real-time PCR
