# Supplementary material for: Circular RNA expression profile in the spinal cord of morphine tolerated rats and screen of putative key circRNAs
Source: Mol Brain. 2019 Sep 18;12:79. doi: 10.1186/s13041-019-0498-4 (PMC6751888; doi:10.1186/s13041-019-0498-4)
Supplement: Supplementary file 1 — Additional file 1: Table S1. The primers for real-time PCR. [file 13041_2019_498_MOESM1_ESM.docx]

Table S1.The primers for real-time PCR

| **gene** | **Prime sequence** | **annealing temperature (℃)** | **Length of PCR product(bp)** |
| --- | --- | --- | --- |
| GAPDH(RAT) | F:5’ GCTCTCTGCTCCTCCCTGTTCTA3'  R:5’ TGGTAACCAGGCGTCCGATA3’ | 60 | 124 |
| rno_circRNA_010774 | F:5' CTGAACAACAAGAATCCCAGAAC3'  R :5’ GCCCGTAAACACATGATACAAAC3’ | 60 | 207 |
| rno_circRNA_005151 | F:5' GGACAAAAATCGGATAATAAGA3'  R :5’ AGAGGACTTTTTCTGATGCTAA3’ | 60 | 184 |
| rno_circRNA_008508 | F:5' GATTCCAATGATGTATCAACAAGG3'  R :5’ AGAGCAGTCCAAGGTAAGCCAT3’ | 60 | 78 |
| rno_circRNA_015657 | F:5' CAGGAAGTATTTTTATTGGAAGAGG3'  R :5’ TCTGGGGGGAAACAAATCATC3’ | 60 | 103 |
| rno_circRNA_012605 | F:5' TACCCCACAAGGTTGCCTGT3'  R :5’ TGCTCTTCCACAGTTCAGTCCA3’ | 60 | 144 |
| rno_circRNA_017999 | F:5' TAGATGCCAACGGGGTCCCT3'  R :5’ TGAGTCTGGGTCATCGGGAAC3’ | 60 | 200 |
| rno_circRNA_004800 | F:5' ACTCCTGGCTAAGCTGGAGG3'  R :5’GGCTGGCCATCTGACTGACT3’ | 60 | 121 |
| rno_circRNA_014599 | F:5' ACCTCAGAAACCTCAGTCAGCA3'  R :5’ AGCATAGCAGGACCACCACT3’ | 60 | 187 |
| rno_circRNA_000047 | F:5' ACCTCAGCCTCAGCAGCAAT3'  R :5’ GGATGGCTGAGGAGGAGCAG3’ | 60 | 171 |
