# Supplementary material for: Circular RNA expression profile in the spinal cord of morphine tolerated rats and screen of putative key circRNAs
Source: Mol Brain. 2019 Sep 18;12:79. doi: 10.1186/s13041-019-0498-4 (PMC6751888; doi:10.1186/s13041-019-0498-4)
Supplement: Supplementary file 2 — Additional file 2: Table S2. The detailed information of top 30 up-regulated and 30 down-regulated circRNAs. [file 13041_2019_498_MOESM2_ESM.docx]

Table S2. The detailed information of top 30 up-regulated and 30 down-regulated circRNAs

| **Up-regulatedcircRNAs** | **Fold change(MT/NS)** | ***P*-value** | **GeneSymbol** | **group-MT(raw)** | **group-NS(raw)** |
| --- | --- | --- | --- | --- | --- |
| mmu_circRNA_36795 | 6.0845975 | 0.018356319 | RGD1564809 | 419.25 | 235.125 |
| rno_circRNA_009458 | 5.8074712 | 0.027309230 | Rictor | 336.75 | 155.75 |
| rno_circRNA_016457 | 5.7272536 | 0.010350693 | Dis3l | 350 | 151.25 |
| rno_circRNA_004155 | 5.7259444 | 0.012519971 | Kdm5b | 261.25 | 116.875 |
| rno_circRNA_001246 | 5.7195934 | 0.020625997 | Fam178a | 255.5 | 113.25 |
| rno_circRNA_013958 | 5.6670224 | 0.021508023 | Ttc7 | 611.25 | 347 |
| rno_circRNA_016296 | 5.6437067 | 0.027365629 | Slc35f2 | 172.75 | 73.625 |
| rno_circRNA_015860 | 5.6400487 | 0.011516258 | Setd2 | 547.625 | 312.625 |
| rno_circRNA_001555 | 5.5110625 | 0.025346605 | Pde10a | 538 | 312.625 |
| rno_circRNA_002750 | 5.4581638 | 0.027657895 | Tlk2 | 263.5 | 125.375 |
| rno_circRNA_001507 | 5.3238053 | 0.013889338 | Scaf8 | 511.25 | 251.875 |
| rno_circRNA_012504 | 5.3151921 | 0.012598287 | Prex2 | 231.125 | 103.75 |
| rno_circRNA_002748 | 5.2250529 | 0.024787052 | Tlk2 | 163 | 74.375 |
| mmu_circRNA_36027 | 5.1529633 | 0.017980549 | Lppr4 | 476.625 | 221.625 |
| rno_circRNA_008508 | 5.1019928 | 0.007004750 | Serpini1 | 671.25 | 311 |
| rno_circRNA_003463 | 5.0880770 | 0.008794891 | Slc46a3 | 976.25 | 449.625 |
| rno_circRNA_007341 | 5.0858725 | 0.011376886 | Csnk1g3 | 183.625 | 86.375 |
| rno_circRNA_017300 | 5.0211465 | 0.018699698 | Fam126b | 370.75 | 186.875 |
| mmu_circRNA_28075 | 5.0060787 | 0.021940609 | Ubac2 | 471.625 | 248.75 |
| rno_circRNA_003841 | 4.9899534 | 0.014017251 | Fry | 535.875 | 269.375 |
| rno_circRNA_002713 | 4.9315080 | 0.016009322 | Eftud2 | 506.5 | 291.625 |
| rno_circRNA_013445 | 4.8211269 | 0.019144936 |  | 782.5 | 481.75 |
| rno_circRNA_003127 | 4.7834488 | 0.035158429 | Cblb | 258 | 120.125 |
| rno_circRNA_005773 | 4.6613685 | 0.021098343 | RGD1561609 | 1973.375 | 543.875 |
| rno_circRNA_012315 | 4.6579601 | 0.013540549 | Frem1 | 340.25 | 213.125 |
| rno_circRNA_006313 | 4.6378870 | 0.020471688 | Fam70b | 768.125 | 391.75 |
| rno_circRNA_004800 | 4.4214763 | 0.011443161 | Evi5 | 271.5 | 151 |
| rno_circRNA_000047 | 2.1238537 | 0.009865310 | Nav2 | 5486.25 | 3387.625 |

| **circRNA** | **FC (abs)** | ***P*-value** | **GeneSymbol** | **group-MT(raw)** | **group-NS(raw)** |
| --- | --- | --- | --- | --- | --- |
| rno_circRNA_005151 | 12.125677 | 0.003497230 | Whsc1 | 150.625 | 1872.375 |
| mmu_circRNA_31675 | 11.094593 | 0.013568066 | Rbm27 | 366.5 | 2927.625 |
| rno_circRNA_005882 | 10.554504 | 0.004136434 | Psd3 | 448.75 | 5631.5 |
| rno_circRNA_005883 | 9.2261508 | 0.003722234 | Psd3 | 394.25 | 4614 |
| rno_circRNA_010774 | 8.8206270 | 0.028672918 | RGD1560248 | 589.75 | 3626.5 |
| rno_circRNA_009194 | 8.7596182 | 0.008853479 | Homer1 | 237.5 | 2436.25 |
| rno_circRNA_017999 | 8.3456462 | 0.044199791 | Zfx | 718.25 | 4113 |
| rno_circRNA_004568 | 8.2347720 | 0.034998365 | Vps54 | 289.25 | 1980.5 |
| rno_circRNA_000819 | 8.1257068 | 0.024806782 | LOC100360606 | 481.875 | 3212.625 |
| rno_circRNA_016943 | 8.0659051 | 0.035785593 | Tbc1d5 | 830.25 | 4731 |
| rno_circRNA_016945 | 7.9787274 | 0.032583911 | Tbc1d5 | 943.625 | 5265.375 |
| rno_circRNA_014598 | 7.9373836 | 0.041628689 | Ralgapa1 | 341.125 | 2382.875 |
| rno_circRNA_013019 | 7.6551661 | 0.042293287 | Rere | 2727.375 | 12837.75 |
| rno_circRNA_009921 | 7.6490638 | 0.037065004 | Hipk3 | 815.625 | 4280.875 |
| rno_circRNA_016441 | 7.6440329 | 0.034586158 | Pias1 | 304.875 | 1757.25 |
| rno_circRNA_016592 | 7.6123436 | 0.014279562 | Vps13c | 161.5 | 1299.375 |
| rno_circRNA_013017 | 7.5584869 | 0.044158163 | Rere | 3135.375 | 14106 |
| rno_circRNA_007241 | 7.5564283 | 0.042352048 | Rbm27 | 292.875 | 1733.5 |
| rno_circRNA_013028 | 7.4860472 | 0.034756576 | Rere | 1693.25 | 9054.5 |
| rno_circRNA_012084 | 7.4618035 | 0.003016445 | Mkln1 | 108.25 | 1549.875 |
| rno_circRNA_016940 | 7.4557175 | 0.049599555 | Tbc1d5 | 1916.125 | 8809.75 |
| rno_circRNA_018001 | 7.4177919 | 0.032649074 | Zfx | 964.375 | 5668.625 |
| rno_circRNA_001879 | 7.2950289 | 0.007445991 | RGD1310862 | 253.5 | 2482.5 |
| rno_circRNA_016225 | 7.2879212 | 0.023932451 | Gucy1a2 | 302.5 | 2002.375 |
| rno_circRNA_015180 | 7.2798523 | 0.010852100 | Anks1b | 187.875 | 1513 |
| rno_circRNA_013018 | 7.2545071 | 0.039660048 | Rere | 2625.5 | 12792.125 |
| rno_circRNA_001129 | 7.1757642 | 0.043360239 | Pten | 350.375 | 2049.875 |
| rno_circRNA_013016 | 7.1359877 | 0.042511205 | Rere | 1722.5 | 7618.625 |
| rno_circRNA_012605 | 6.1070689 | 0.003661929 | Nrd1 | 119.625 | 967 |
| rno_circRNA_014599 | 5.7256954 | 0.010650918 | Ralgapa1 | 233.25 | 1604.5 |
