# Supplementary figures and images for: Circular RNA expression profile in the spinal cord of morphine tolerated rats and screen of putative key circRNAs
Source: Mol Brain. 2019 Sep 18;12:79. doi: 10.1186/s13041-019-0498-4 (PMC6751888; doi:10.1186/s13041-019-0498-4)

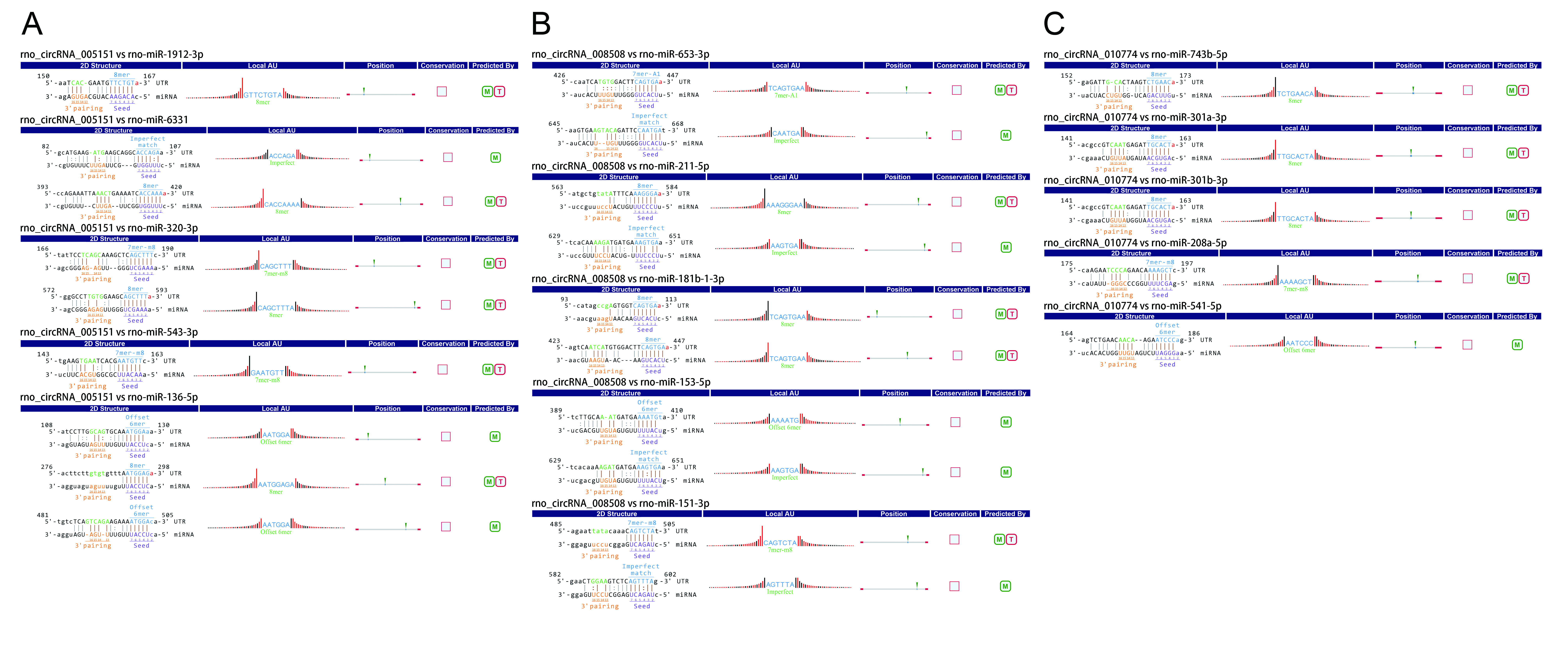

Supplement: Supplementary file 3 — Additional file 3: Figure S1. The putative target miRNAs of the validated circRNAs with the highest matching score. A-C. Putative target miRNAs of circRNA _ 005151, _008508 and _010774 respectively. [file 13041_2019_498_MOESM3_ESM.tif]
